# Caveats of assessing incidence trends using publicly available registry data

**DOI:** 10.1007/s10654-025-01288-9

**Published:** 2025-09-04

**Authors:** Volker Arndt, Soo-Zin Kim-Wanner, Bernd Holleczek, Alice Nennecke, Frederik Peters, Annika Waldmann

**Affiliations:** 1 Baden-Württemberg Cancer Registry & Unit of Cancer Survivorship, German Cancer Research Center: Deutsches Krebsforschungszentrum, Heidelberg, Germany; 2 Hessian Cancer Registry, Hessian Office of Health and Care, Frankfurt am Main, Germany; 3 Saarland Cancer Registry, Saarbrücken, Germany; 4 Hamburg Cancer Registry, Hamburg, Germany; 5 Institute for Social Medicine and Epidemiology, University of Lübeck, Lübeck, Germany

We read with great interest the article “Trends in incidence and mortality of early-onset cancer in Germany between 1999 and 2019” by Voeltz et al. published in the European Journal of Epidemiology [1, 2]. Although the approach to use secondary data is attractive, there are several limitations which need to be raised in our view.

Firstly, estimates of cancer incidence for Germany were based on a varying composition of population-based cancer registries and populations.

The data used for the analysis by Voeltz et al. were estimates (projections) rather than purely observed counts. As shown in Supplement S6, the completeness of the cancer registries has varied according to study period and region. As a consequence, the annual national estimates published by Centre for Cancer Registry Data (ZfKD) are based on a varying set of included population-based state registries with different background cancer risk over time. For example, coverage in the early years was limited to incidence data of only 7 registries, whereas 15 registries contributed directly to the estimates in more recent years. The annual incidence data for those states, which were not part of the composition to derive nationwide incidence estimates in a specific year, were based on incidence/mortality ratios and observed state wide cancer mortality. Some states with lower overall cancer incidence (such as Baden-Württemberg) were included only in recent years. While the incidence estimates for earlier years consider such state-specific variation, they remain nonetheless more uncertain than those for more recent years. This may introduce bias that is particularly relevant for estimates of rare cancers, for example pertaining to early-onset colorectal cancer, and that becomes visible when trends in cancer incidence based on the national estimates are compared with trends derived from a fixed pool of registries [3].

Secondly, overestimation of population figures in more recent years.

The Federal Statistical Office of Germany informed on June 25, 2024 that, as of May 15, 2022, the population figure based on the official intercensal population update of the 2011 census has overestimated the population in Germany by about 1.4 million people (1.8%) when compared with the 2022 census. The degree of overestimation of the population figures is assumed to have continuously increased over the years. Above-average differences in the population figures were recorded for the foreign population, in large municipalities, for young adults and for centenarians [4, 5]. As a consequence, times series of incidence rates based on 2011 census population data underestimate trends in cancer incidence. However, detailed population data required for proper calculation of incidence rates after the year 2011 have not yet been published as of June 2025.

Taken together these issues likely resulted in an underestimation of cancer incidence and the corresponding trend in the later years of the study period. The national data published by ZfKD represent a valuable and important resource to inform the public about the burden of cancer in Germany. However, these estimates should not be used for scientific analysis. Instead, we recommend to use record-level data pertaining to selected states which can be requested either directly from the ZfKD or from the individual registries. This approach would enable trend analysis based on a fixed set of registers using predefined quality criteria and consultation with data holders regarding the suitability of the requested data for specific analyses.

Volker Arndt (Baden-Württemberg Cancer Registry & Unit of Cancer Survivorship, German Cancer Research Center), Soo-Zin Kim-Wanner (Hessian Cancer Registry), Bernd Holleczek (Saarland Cancer Registry), Alice Nennecke & Frederik Peters (Hamburg Cancer Registry), Annika Waldmann (Institute for Social Medicine and Epidemiology, University of Lübeck) for the ALSTER working group (Age-specific trends in cancer incidence in Germany).
